# Phase II study of preoperative radiation plus concurrent daily tegafur-uracil (UFT) with leucovorin for locally advanced rectal cancer

**DOI:** 10.1186/1471-2407-11-98

**Published:** 2011-03-16

**Authors:** Patrice Cellier, Bernard Leduc, Laurent Martin, Brigitte Vié, Christian Chevelle, Véronique Vendrely, Augustin Salemkour, Christian Carrie, Gilles Calais, Pascal Burtin, Loïc Campion, Michèle Boisdron-Celle, Alain Morel, Virginie Berger, Erick Gamelin

**Affiliations:** 1 Centre Paul Papin, 2 rue Moll, 49933 Angers cedex 9, France; 2 Centre Hospitalier, Boulevard d DocteurVerlhac, 19312 Brive la Gaillarde Cedex, France; 3 Centre Guillaume le Conquérant, 29 rue Guillaume le Conquérant, 76600 Le Havre, France; 4 Centre François Baclesse, Avenue du Général Harris, BP 5026, 14 07 Caen Cedex 5, France; 5 Clinique Pasteur, 45 avenue de Lombez, BP 27617, 31076 Toulouse Cedex 3, France; 6 Groupe Hospitalier Saint-André, 1 rue Jean Burguet, 33000 Bordeaux, France; 7 Centre d’Onco-Radiothérapie d’Eure et Loire, 4 rue Claude Bernard (site Fontenoy), B.P. 10309 28600 Chartres, France; 8 Centre Léon Bérard, 28 rue Laennec, 69373 Lyon Cedex 8, France; 9 CHU Bretonneau, CORAD 2 Boulevard Tonnelé, 37044 Tours Cedex, France; 10 Centre Hospitalier Universitaire, 4 rue Larrey, 49933 Angers Cedex 9, France; 11 Centre René Gauducheau, Bd Jacques Monod, 44805 Nantes St Herblain Cedex, France; 12 CRCNA INSERM U892, 1, quai de Tourville BP 13522, 44035 Nantes Cedex 1, France

## Abstract

**Background:**

Considerable variation in intravenous 5-fluorouracil (5-FU) metabolism can occur due to the wide range of dihydropyrimidine dehydrogenase (DPD) enzyme activity, which can affect both tolerability and efficacy. The oral fluoropyrimidine tegafur-uracil (UFT) is an effective, well-tolerated and convenient alternative to intravenous 5-FU. We undertook this study in patients with locally advanced rectal cancer to evaluate the efficacy and tolerability of UFT with leucovorin (LV) and preoperative radiotherapy and to evaluate the utility and limitations of multicenter staging using pre- and post-chemoradiotherapy ultrasound. We also performed a validated pretherapy assessment of DPD activity and assessed its potential influence on the tolerability of UFT treatment.

**Methods:**

This phase II study assessed preoperative UFT with LV and radiotherapy in 85 patients with locally advanced T3 rectal cancer. Patients with potentially resectable tumors received UFT (300 mg/m/^2^/day), LV (75 mg/day), and pelvic radiotherapy (1.8 Gy/day, 45 Gy total) 5 days/week for 5 weeks then surgery 4-6 weeks later. The primary endpoints included tumor downstaging and the pathologic complete response (pCR) rate.

**Results:**

Most adverse events were mild to moderate in nature. Preoperative grade 3/4 adverse events included diarrhea (n = 18, 21%) and nausea/vomiting (n = 5, 6%). Two patients heterozygous for dihydropyrimidine dehydrogenase gene (*DPYD*) experienced early grade 4 neutropenia (variant IVS14+1G > A) and diarrhea (variant 2846A > T). Pretreatment ultrasound TNM staging was compared with postchemoradiotherapy pathology TN staging and a significant shift towards earlier TNM stages was observed (p < 0.001). The overall downstaging rate was 42% for primary tumors and 44% for lymph nodes. The pCR rate was 8%. The sensitivity and specificity of ultrasound for staging was poor. Anal sphincter function was preserved in 55 patients (65%). Overall and recurrence-free survival at 3 years was 86.1% and 66.7%, respectively. Adjuvant chemotherapy was administered to 36 node-positive patients (mean duration 118 days).

**Conclusion:**

Preoperative chemoradiotherapy using UFT with LV plus radiotherapy was well tolerated and effective and represents a convenient alternative to 5-FU-based chemoradiotherapy for the treatment of resectable rectal cancer. Pretreatment detection of DPD deficiency should be performed to avoid severe adverse events.

## Background

The standard approach for the preoperative treatment of rectal cancer is intravenous (i.v.) 5-fluorouracil (5-FU)-based chemoradiotherapy [1-3] (Rich *et al*, 1995; Lawrence *et al*, 1997; Bosset *et al *2000). 5-FU can be administered as either a bolus injection or a continuous infusion [4] [Smalley 2006]. However, continuous infusion requires specialized pumps, which are inconvenient for patients, and long-term venous access, which makes patients susceptible to infections and thrombosis [1,2] (Rich *et al*, 1995; Bosset *et al*, 2000).

The oral fluoropyrimidine UFT (tegafur-uracil) is an effective, well-tolerated and convenient alternative to i.v. 5-FU that is widely used in the treatment of colorectal cancer [5] (Borner *et al*, 2002). A phase I study of preoperative UFT with leucovorin (LV) with radiotherapy identified a maximum tolerated dose of UFT of 350 mg/m^2^/day with LV 90 mg/day [6] (Hoff *et al*, 2000). In phase II and III trials, UFT (200-350 mg/m^2^/day) without or with LV (15-45 mg/day) and preoperative radiotherapy (45-50.4 Gy) has shown similar tumor response and downstaging rates to those using continuous infusion 5-FU [7-14] (de la Torre *et al*, 1999, de la Torre *et al*, 2008 Feliu *et al*, 2002; Wang *et al*, 2005; Fernández-Martos *et al*, 2004; Girault 2008; Kundel 2007; Vestermark 2008).

Considerable variation in 5-FU metabolism and outcome due to the wide range of dihydropyrimidine dehydrogenase (DPD) enzyme activity has been observed and this variability can influence both tolerability and response to cancer chemotherapy [15] (Seck *et al*, 2005). Although deficient DPD activity is rare (approximately 3-5% of patients), patients with this deficiency may experience severe and life-threatening adverse events following treatment with 5-FU or oral fluoropyrimidines such as capecitabine [16-18] (Diasio and Johnson, 1999; Van Kuilenburg *et al*, 2003; Saif and Diasio, 2006).

We undertook the present multicenter study in patients with locally advanced rectal cancer to evaluate the efficacy and tolerability of UFT with LV and preoperative radiotherapy and to evaluate the utility and limitations of multicenter staging using pre- and post-chemoradiotherapy ultrasound. Since little information is available regarding DPD activity in UFT-treated patients with rectal cancer, we also performed a validated pretherapy assessment of DPD activity and assessed its potential influence on the tolerability of UFT treatment.

## Methods

### Patients

Eligible patients had pathologically confirmed clinically resectable stage T3-T4 and N0-N2 adenocarcinoma of the distal rectum with no distant metastases. Entry criteria were: inferior margin within 15 cm of the anal verge and palpable on digital examination; World Health Organization (WHO) performance status 0-2; age 18-80 years; adequate hematologic (neutrophils >1500/mm^3^), hepatic (bilirubin <2 upper normal limit), and renal (creatinine <150 μmol/L) function. Patients with prior malignancies (excluding localized epithelial skin or cervical cancer), chemotherapy, and/or pelvic radiotherapy were excluded. The study was approved by local ethics committees and was conducted according to the Declaration of Helsinki. Written informed consent was obtained from each patient before enrollment.

Pretreatment evaluation included a complete medical history and physical examination (including digital rectal examination), complete blood count and chemistry profile, electrocardiogram, detection of DPD activity (requiring a 10 days period), endorectal ultrasound (EUS), endoscopy with biopsy, chest X-ray, and abdomen-pelvis computed tomography (CT) scan.

### Treatment

Chemotherapy consisted of UFT 300 mg/m^2^/day and LV 75 mg/day orally in three divided doses taken at approximately 8-hour intervals (either 1 hour before or at least 1 hour after food), 5 days/week for 5 weeks (days 1-33). Pelvic radiotherapy (daily dose 1.8 Gy), delivered concurrently with chemotherapy, was delivered using a conventional four-field box technique (anterior and posterior fields, plus right and left lateral fields) and with a minimum of 10 megavoltage photons. A total dose of 45 Gy was delivered to the isocenter of the four fields. The planning target volume was defined as the clinical target volume, i.e. the primary tumor, iliac lymph nodes, and mesorectum *in toto*, plus a 10-15 mm margin. Diagnostic imaging was used to define the gross target volume.

Surgical resection was performed 4-6 weeks after completion of chemoradiotherapy using a standardized technique with mesorectal excision. Postoperative chemotherapy could be prescribed for patients who were lymph-node positive and who responded to preoperative treatment. These patients received 5 cycles of the same UFT with LV regimen or 5-FU/LV (de Gramont or Mayo clinic regimens) 3-6 weeks after surgery.

### Toxicity assessment and dose adjustments

Toxicity, graded using WHO criteria for chemotherapy and early radiotherapy toxicity, was assessed at weekly clinic and biologic examinations during chemoradiotherapy and at the end of treatment [19] (Miller *et al*, 1981). Late radiotherapy toxicity was graded according to the SOMA-LENT criteria [20] (Mornex *et al*, 1997). In the event of grade 2-4 adverse events, chemotherapy was withheld until evidence of hematologic recovery (neutrophils ≥ 1500/mm^3 ^and platelets ≥100 000/mm^3^) and complete resolution of all nonhematologic adverse events (other than alopecia) to baseline or grade 1. The UFT dose was reduced by one capsule per day if grade 3/4 toxicity occurred. Radiotherapy was postponed for 1-2 weeks in the event of grade 3 diarrhea that persisted despite UFT discontinuation; radiotherapy was stopped if the grade 3/4 diarrhea persisted for longer than 2 weeks.

### Evaluation of response

EUS, chest X-ray, and CT scan were performed at baseline (within 15 days before chemoradiotherapy) and after the end of chemoradiotherapy (within 7 days before surgery). Echoendoscopic and pathologic stages were defined using the tumor node metastasis (TNM) staging system (version 5.0) [21] (Sobin and Wittekind, 1997). Initially, it was planned to determine tumor downstaging by comparing posttreatment ultrasound (u)TNM stage (EUS) with the preoperative uTNM stage. However, due to the limitations of preoperative EUS because of inflammatory effects and tissue modifications and the limited number of assessable patients, we focused on pathological results and compared posttreatment pathological (p)TNM stage to preoperative uTNM stage. Primary tumor and node downstaging were defined as reductions in T and N stages by at least one level. No response was defined as similar pTNM and uTNM classifications. A pCR was defined as the absence of any residual tumor cells in the operative specimen [22] (Meterissian *et al*, 1994). Lymph nodes were examined after treatment with a clarification solution in order to aid detection and improve the reliability of the pathologic N staging. All resected specimens from the different hospitals were centralized and reexamined in a reference pathology laboratory for a central review.

Sphincter function was scored according to the Memorial Sloan Kettering Cancer Center scale [23] (Minsky *et al*, 1995). Progression-free survival (PFS) and overall survival (OS) were measured from the time of inclusion to the time of disease progression and death (or last follow-up), respectively.

### Detection of DPD activity

*DPYD *genotyping included examination of 24 relevant gene variants potentially involved in DPD deficiency. Single nucleotide polymorphisms (SNPs) were detected using a real-time mini-sequencing method [24] (Morel *et al*, 2005). Dihydrouracil and uracil plasma concentrations were determined simultaneously using a liquid chromatography method [25] (Remaud *et al*, 2005); if DPD deficiency was suspected, the plasma dihydrouracil:uracil (UH_2_:U) ratio was determined [26] (Boisdron-Celle *et al*, 2007). UH_2_:U ratios were assessed as previously described [27] (Gamelin *et al*, 1999). When DPD deficiency was identified, UFT was administered at the planned dose but both the clinician and patient were informed of the risk associated with treatment and appropriate precautions were taken, such as careful monitoring of diarrhea and neutropenia.

### Statistics

Using a two-stage design [28] (Simon, 1989) and assuming a tumor downstaging rate of at least 25%, with alpha and beta errors of 5% and 10%, respectively, 41 patients were to be enrolled in the first stage of the study. If four or fewer responses were observed, the treatment would be considered ineffective and the trial would be stopped. If five or more responses were observed, 29 additional patients would be enrolled, for a total of 70 patients. As numerous patients were not assessable for EUS, 15 additional patients were recruited with the approval of IERB.

The primary endpoint was the tumor downstaging rate with 95% confidence interval (CI). Results are expressed as mean ± standard deviation. Quantitative variables were compared using the nonparametric Mann-Whitney test and qualitative variables using Pearson's chi-squared test or Fisher's exact test. Survival distributions were estimated using the Kaplan-Meier methodology. For safety analyses, the worst toxicity grade per patient in all chemotherapy cycles was used. All statistical analyses were conducted using Statistical Package for Social Sciences software (SPSS version 10.0, Chicago, IL, USA).

## Results

Eighty-five patients were enrolled at 15 centers between July 2002 and January 2004. Eight patients were ineligible at study entry: four patients had one or more major protocol violations (liver metastases, n = 3; suspicion of pulmonary metastases, n = 1; other cancer, n = 1) and six patients had one or more minor protocol violations (tumor in the upper rectum, n = 4; age >80 years, n = 1; vesical papilloma, n = 1). All 85 patients were evaluated according to the intention-to-treat principle.

### Patient characteristics

Patient characteristics at baseline are shown in Table 1. Four of the 24 known variants of the *DPYD *gene were identified. One patient was heterozygous for the -1590T > C SNP in the promoter region, one was heterozygous for IVS14+1G > A (G1A [DPYD*2A]), one had 2846A > T (D949V), and a fourth had 1679 T > G. A further 38 patients were heterozygous for 85 T > C. Uracil plasma levels were higher than the threshold (15 μg/L) [26] (Boisdron-Celle *et al*, 2007) in three of four patients with variant *DPYD *(IVS14+1G > A: 36 μg/L; 2846A > T: 19 μg/L; and 1679 T > G: 25 μg/L).

**Table 1:** Baseline patient characteristics

Parameter	Number of patients (%) N = 85
**Age (years)**	
Median	67.1
Range	25-81
**Gender**	
Male	56 (66)
Female	29 (34)
**Clinical TNM stage**	
T3	85 (100)
N0	47 (55)
N1	33 (39)
N2	2 (2)
Unknown	3 (4)
**Tumor staging method**	
EUS	66 (78)
Rigid rectoscopy	11 (13)
Other	7 (8)
Unknown	1 (1)
**Distance from anal verge**	
>5 cm	50 (59)
≤5 cm	32 (38)
Unknown	3 (4)
**WHO performance status**	
0	75 (88)
1	9 (11)
Unknown	1 (1)

TNM = tumor node metastasis

WHO = World Health Organization

### Treatment

Sixty-three patients (74%) completed treatment as planned. Most patients (92%) received the planned dose of radiotherapy (median dose 45 Gy, range 18-46.8 Gy). Radiotherapy was postponed for at least 1 day in 66 patients (78%); dose reductions were drug related in five patients (6%). The planned UFT dose was delivered in 48 patients (mean dose 294 ± 24 mg/m^2^). Chemotherapy was delayed in nine patients (11%) and the dose was reduced in 15 patients (18%) as a result of adverse events, including diarrhea (n = 5), nausea/vomiting (n = 1), stroke (n = 1), dehydration (n = 3), and gastrointestinal effects (n = 1). Only four (5%) of the 85 patients did not undergo surgery: one patient died of a cerebrovascular stroke (unrelated to treatment) during the preoperative period; one patient was lost to follow-up; and the primary tumor was unresectable in two patients because of local progression. The majority of patients underwent anterior resection (AR) (n = 54; 63%); abdominoperineal resection (APR) was necessary in 27 patients (32%).

### Safety

Diarrhea was the most common adverse event (60 events, 42 of which were judged to be treatment related). Most adverse events were mild to moderate in nature. Grade 3/4 adverse events, which occurred in 38 patients (45%), are shown in Table 2. Only patients who were heterozygous for variants IVS14+1G > A and 2846A > T experienced very early grade 4 neutropenia and diarrhea related to UFT. The patient who was heterozygous for variant 1679 T > G experienced grade 4 diarrhea related to treatment at week 3 and was withdrawn.

**Table 2:** Acute adverse events that occurred during preoperative chemoradiotherapy

	Number of patients (%) N = 85	
**Adverse event**	**Grade 3**	**Grade 4**

Diarrhea	14 (17)	4 (5)
Nausea/vomiting	4 (5)	1 (1)
Cerebrovascular event	0	2 (2)
Proctitis	1 (1)	0
Radiocystitis	1 (1)	0
Radiodermatitis	2 (2)	0
Anemia	1 (1)	0
Leukopenia	0	1 (1)
Neutropenia	0	1 (1)
Hand-foot syndrome	0	0
Mucositis	1 (1)	0
Subocclusive syndrome	1 (1)	0
Second-degree cutaneous burns*	1 (1)	0
Epistaxis	1 (1)	0
Pneumopathy	1 (1)	0
Thrombophlebitis	1 (1)	0
Total	29 (34)	8 (9)

1. *Not related to treatment

### Downstaging, pathologic response, and sphincter preservation

EUS was available pre- and posttreatment for 66 patients and was interpretable in 52 evaluable patients (61%). Postchemotherapy uTN staging was in agreement with double-checked pTN staging in 26 of 52 patients (50%) (Table 3). Pretreatment uTNM staging was compared with postchemoradiotherapy pTN staging (Table 4) and a significant shift towards earlier TNM stages was observed (p < 0.001). Downstaging of the primary tumor occurred in 22/52 patients (42%) and downstaging of the lymph nodes occurred in 23/52 patients (44%). In the 46 patients assessable for T and N stage, 11 patients (24%) had downstaged tumor and nodes, 11 patients (24%) had downstaged nodes only, nine patients (20%) had downstaged tumor only, and there was no difference in T or N staging in 15 patients (33%). The overall downstaging rate was 44% for primary tumors and 48% for lymph nodes. A centralized second pathologic examination performed in 78 patients confirmed the initial pTN results in 77% of cases. For nine patients, the first pathologic examination was understaged (four pT0, three pT1, two pT2, and one pT3), and for three patients it was overstaged (two pT2 and one pT4). Two patients with nonspecified T stage were classified pT3. In seven patients, malignant cells were undetectable in both the primary tumor and lymph nodes giving a pCR rate of 8%. Ten patients (12%) had some microscopic residual disease (Tmic).

**Table 3:** Post-treatment EUS T staging and pathologic analysis T staging

		Pathologic analysis			
**Number of patients (%)**		**T0**	**T2**	**T3**	**Total**

EUS	T0	3 (43)	-	4 (57)	7
	T1	1 (50)	-	1 (50)	2
	T2	3 (20)	4 (27)	8 (53)	15
	T3	-	8 (30)	19 (70)	27
	Total	7	12	32	51

EUS = endorectal ultrasonography

**Table 4:** Pre-treatment EUS T and N staging and post-treatment pathologic T and N staging in resected patients

**Pre-treatment**	**Post-treatment pathologic stage**				**Downstaging, n/N (%)**
	
**EUS T stage**	**pT0**	**pT1**	**pT2**	**pT3**	
T2*	1	0	2	0	1/3 (33)
T3	6	2	13	28	21/49 (43)
Total	7	2	15	28	22/52 (42)
**EUS N stage**	**pN0**	**pN1**			
N0	19	0			0/19
N1	22	10			22/32 (69)
N2	0	1			1/1 (100)
Total	41	11			23/52 (44)

EUS = endorectal ultrasonography

*Three patients had an estimated T2 tumor using EUS but had a T3 tumor identified clinically

Anal sphincter function was preserved in 55 patients (65%). Five of 60 patients initially deemed suitable for anterior resection (AR) had an abdominoperineal resection (APR) (8% of planned resections) and two of 23 patients whose planned surgery was APR underwent an AR (9% of planned resections). APR was performed in 60% of patients (n = 21) with a tumor in the distal third of the rectum and in 9% of patients (n = 4) with a tumor in the middle third of the rectum (within 6-8 cm of the pectineal line) due to an overestimated distance to the anal verge and to infiltration of the surgical border. AR was performed in 87% of patients (n = 41) with a tumor in the middle third of the rectum and in 40% of those whose tumor was in the distal third (n = 14) (unknown pectineal line n = 2, inextirpable n = 2). Perioperative and 30-day postoperative complications included anastomotic leakage (n = 1), perineal complications (n = 1), bleeding (n = 1), ileus (n = 1), fistula (n = 1), and death (n = 2).

### Survival and recurrence

The mean follow-up time was 45 months (range 1 - 84 months; one patient was lost to follow-up). OS was 92.6% at 1 year 86.1% at 2 years and 83.2% at 3 years; mean recurrence-free survival was 39 ± 24 months. Recurrence free survival was 81.9% at 1 year, 70.7% at 2 years and 66.7% at 3 years.

Two patients died within 18 days of surgery (one had a pulmonary embolism and the other had a cerebrovascular stroke). Sixteen other patients died during the study: twelwe due to progressive disease; one due to diabetic decompensation, one due to vascular cerebral stroke that occurred before surgery, and one due to acute pulmonary failure and one due to a fall.

The level of local control in the 81 patients who underwent surgery was 95%, with four patients (5%) having local recurrence and 20 (24.7%) having secondary metastases. Regardless of location, relapse generally occurred <1 year after surgery (mean: 346 ± 236 days).

### Adjuvant chemotherapy

Following surgery, 36 of 79 eligible patients (46%) received adjuvant chemotherapy (mean duration 118 ± 65.8 days): 21 patients (58%) received 5-FU-based chemotherapy, 10 (28%) received UFT-based chemotherapy and 4 (11) received oxaliplatin-based chemotherapy. Six patients had further chemotherapy for metastatic disease within 4 months of surgery.

## Discussion

Preoperative radiotherapy with or without i.v. 5-FU is a reference treatment for patients with rectal cancer. The aim of the present study was to evaluate the efficacy and tolerability of the combination of UFT-based chemotherapy and radiotherapy before resection. We found a significant downstaging rate (44% for primary tumors and 48% for lymph nodes) similar to capecitabine [29] (Kim *et al*, 2007). The pCR rate was 8%, which is similar to that previously reported [11,30,31] (Fernández-Martos *et al*, 2004; Sauer *et al*, 2004; Durnst *et a*l, 2008). The sphincter preservation rate was also high (65%). The 2-year OS rate was 80%, with 75% of patients free from recurrence, and the rate of local relapse was 5%. These results are similar to those obtained in other trials, many of which used more aggressive regimens, such as combination oxaliplatin and fluoropyrimidine-based chemoradiotherapy [32-34] (Sebag-Montefiore *et al*, 2005; Calvo *et al*, 2006; Ryan *et al*, 2006).

Although the primary objective of this study was to determine the downstaging of tumor and lymph nodes by comparing EUS before and after chemoradiotherapy, we decided to compare the initial EUS to pathological findings. The accuracy of EUS, which was mandatory for pretreatment evaluation in our study, has been reported to range from 62-66% for the assessment of rectal wall penetration and 23% for the determination of nodal status [35,36] (Janjan *et al*, 1999; Chan *et al*, 2005). However, EUS is not possible in circumferential tumors with stenosis and it is generally difficult to interpret 6 weeks after completion of chemoradiotherapy. In addition, EUS is highly dependent on the operator and consistency of results can be an issue in multicenter trials. The nonspecific inflammatory effects and deep alterations evident in tissue architecture after chemoradiotherapy make this examination useless after treatment, despite its importance in determining the extent of the tumor before surgery. Thus, we compared the initial echoendoscopy with the pathologic exam [37,38] (Chan *et al*, 2005; Mawdsley *et al*, 2005). Sensitivity and specificity were not good even at the first pretreatment examination: some tumors initially staged as T0 or T1 by EUS were found to be T3 on pathologic examination. Based on evidence from the literature and our own experience, the current practice in our institutions is to use magnetic resonance imaging for the evaluation of local and regional tumor extension, as it provides more precise and reliable results [38,39] (Rasheed *et al*, 2006; Muthusamy *et al*, 2007).

The tolerability of the UFT chemoradiotherapy was acceptable and diarrhea, as expected, was the most common adverse event. Two patients with heterozygous variants (IVS14+1G > A and 2846A > T) plus the phenotype of major enzyme deficiency experienced grade 4 neutropenia and diarrhea. At the time this study was undertaken, no data were available to link specific DPD deficiencies and severe UFT-related adverse events. Therefore, no dose adjustments were specified in the protocol for patients with particular gene variations, and two patients had grade 4 adverse events that might have been avoided. Armed with this information, however, we now systematically test for DPD by both DPD gene variants and UH_2_:U ratio before beginning UFT-based treatment and reduce the UFT dose in patients with major enzyme deficiencies as we do for all i.v. and oral fluoropyrimidine therapy [26] (Boisdron-Celle *et al*, 2007).

The pattern of relapse in patients with locally advanced rectal cancer and the tolerability of treatment [37,38] (Mawdsley *et al*, 2005; Rasheed *et al*, 2006) call into question the approach to preoperative intensification of chemoradiotherapy currently under investigation elsewhere. In our study, only 5% of patients experienced a local relapse similar to that observed by other authors [40,41] (Gérard *et al*, 2006; Bosset *et al*, 2006).

On the other hand, 15% of the patients died due to distant secondary metastases. Thus, the challenge that remains following chemoradiotherapy for locally advanced rectal cancer is to control distant metastases. In colon cancer, the risk of distant metastasis is reduced with adjuvant chemotherapy and this might also apply to the rectal cancer setting. Many authors have emphasized the negative impact of neoadjuvant chemoradiotherapy toxicity on compliance with and tolerability of adjuvant chemotherapy [42,43] (Rodríguez-Ramírez *et al*, 2006; Urso *et al*, 2006). In our study, the mean duration of adjuvant chemotherapy was 4 months, somewhat less than the 6 months usually planned for other tumors, even though UFT has a favorable tolerability profile, better than the other combinations usually reported. Our opinion is that patients would gain more benefit from an efficient adjuvant or perhaps neoadjuvant combination therapy than an intensification of chemoradiotherapy by adding a cytotoxic drug to a fluoropyrimidine [44,45] (Chen *et al*, 2007; Bosset *et al*, 2008). Interest in preoperative chemoradiotherapy for patients with locally advanced rectal cancer is due to the potential for tumor downstaging, which increases the likelihood of curative surgery and allows sphincter preservation in many patients with low-lying tumors. UFT has the advantage of convenient oral administration and a very good tolerability profile, with no hand-foot syndrome.

The efficacy and tolerability of UFT-based chemoradiotherapy reported here are comparable with those previously reported for other fluoropyrimidines and much better than those reported with cytotoxic drug combinations. In addition, detection of DPD deficiency, which is legally recommended for all fluoropyrimidines, has the potential to allow identification of patients with clinically relevant enzyme deficiencies and prevent severe acute adverse events related to this condition.

## Conclusion

Although EUS post chemoradiotherapy failled to assess downstaging, preoperative UFT is an effective, well tolerated, and convenient treatment option for patients with locally advanced rectal cancer.

## Competing interests

The authors declare that they have no competing interests.

## Authors' contributions

PC, BL, LM, BV, CC, VV, AS, CC, GC, PB enrolled patients in the trial PC, VB, EG conceived of the study, and participated in its design and coordination. LC participated in the study design and performed the statistical analysis. MBC, AM carried out the molecular and genetic studies. VB drafted the manuscript. All authors read and approved the final manuscript

## Pre-publication history

The pre-publication history for this paper can be accessed here:

http://www.biomedcentral.com/1471-2407/11/98/prepub

## References

[B1] Rich TA Skibber JM Ajani JA Buchholz DJ Cleary KR Dubrow RA Levin B Lynch PM Meterissian SH Roubein LD Ota DM Preoperative infusional chemoradiation therapy for stage T3 rectal cancer Int J Radiat Oncol Biol Phys 1995 32 1025 9 10.1016/0360-3016(95)00152-O 7607922

[B2] Bosset JF Magnin V Maingon P Mantion G Pelissier EP Mercier M Chaillard G Horiot JC Preoperative radiochemotherapy in rectal cancer: long-term results of a phase II trial Int J Radiat Oncol Biol Phys 2000 46 323 7 10.1016/S0360-3016(99)00411-3 10661338

[B3] Lawrence TS Tepper JE Blackstock AW Fluoropyrimidine-radiation interactions in cells and tumors Semin Radiat Oncol 1997 7 260 6 10.1016/S1053-4296(97)80024-0 10717223

[B4] Smalley SR Benedetti JK Williamson SK Robertson JM Estes NC Maher T Fisher B Rich TA Martenson JA Kugler JW Benson AB Haller DG Mayer RJ Atkins JN Cripps C Pedersen J Periman PO Tanaka MS Jr Leichman CG Macdonald JS Phase III trial of fluorouracil-based chemotherapy regimens plus radiotherapy in postoperative adjuvant rectal cancer: GI INT 0144 J Clin Oncol 2006 24 3542 7 10.1200/JCO.2005.04.9544 16877719

[B5] Borner MM Schöffski P de Wit R Caponigro F Comella G Sulkes A Greim G Peters GJ van der Born K Wanders J de Boer RF Martin C Fumoleau P Patient preference and pharmacokinetics of oral modulated UFT versus intravenous fluorouracil and leucovorin: a randomised crossover trial in advanced colorectal cancer Eur J Cancer 2002 38 349 58 10.1016/S0959-8049(01)00371-9 11818199

[B6] Hoff PM Janjan N Saad ED Skibber J Crane C Lassere Y Cleary KR Benner S Randolph J Abbruzzese JL Pazdur R Phase I study of preoperative oral uracil and tegafur plus leucovorin and radiation therapy in rectal cancer J Clin Oncol 2000 18 3529 34 11032595 10.1200/JCO.2000.18.20.3529

[B7] de la Torre A Ramos S Valcárcel FJ Candal A Regueiro CA Romero J Magallón R Salinas J de las Heras M Veiras C Tisaire JL Aragón G Phase II study of radiochemotherapy with UFT and low-dose oral leucovorin in patients with unresectable rectal cancer Int J Radiat Oncol Biol Phys 1999 45 629 34 10.1016/S0360-3016(99)00225-4 10524415

[B8] de la Torre A García-Berrocal MI Arias F Mariño A Valcárcel F Magallón R Regueiro CA Romero J Zapata I de la Fuente C Fernández-Lizarbe E Vergara G Belinchón B Veiras M Molerón R Millán I Preoperative chemoradiotherapy for rectal cancer: randomized trial comparing oral uracil and tegafur and oral leucovorin vs. intravenous 5-fluorouracil and leucovorin Int J Radiat Oncol Biol Phys 2008 70 102 10 10.1016/j.ijrobp.2007.05.068 17869446

[B9] Feliu J Calvillo J Escribano A de Castro J Sánchez ME Mata A Espinosa E García Grande A Mateo A González Barón M Neoadjuvant therapy of rectal carcinoma with UFT-leucovorin plus radiotherapy Ann Oncol 2002 13 730 6 10.1093/annonc/mdf116 12075741

[B10] Wang LW Yang SH Lin JK Lin TC Chan WK Chen WS Wang HS Jiang JK Lee RC Li AF Chao Y Chi KH Yen SH Preoperative chemoradiotherapy with oral tegafur-uracil and leucovorin for rectal cancer J Surg Oncol 2005 89 256 64 10.1002/jso.20168 15726610

[B11] Fernández-Martos C Aparicio J Bosch C Torregrosa M Campos JM Garcera S Vicent JM Maestu I Climent MA Mengual JL Tormo A Hernandez A Estevan R Richart JM Viciano V Uribe N Campos J Puchades R Arlandis F Almenar D Preoperative uracil, tegafur, and concomitant radiotherapy in operable rectal cancer: a phase II multicenter study with 3 years' follow-up J Clin Oncol 2004 22 3016 22 15210740 10.1200/JCO.2004.11.124

[B12] Vestermark LW Jacobsen A Qvortrup C Vestermark LW Jacobsen A Qvortrup C Hansen F Bisgaard C Baatrup G Rasmussen P Pfeiffer P Long-term results of a phase II trial of high-dose radiotherapy (60 Gy) and UFT/l-leucovorin in patients with non-resectable locally advanced rectal cancer (LARC) Acta Oncol 2008 47 428 33 10.1080/02841860701798866 18348002

[B13] Kundel Y Brenner B Symon Z Oberman B Sadezki S Koller M Catane R Pfeffer R A phase II study of oral UFT and leucovorin concurrently with pelvic irradiation as neoadjuvant chemoradiation for rectal cancer Anticancer Res 2007 27 2877 80 17695464

[B14] Giralt J Tabernero J Navalpotro B Capdevila J Espin E Casado E Mañes A Landolfi S Sanchez-Garcia JL de Torres I Armengol M Pre-operative chemoradiotherapy with UFT and Leucovorin in patients with advanced rectal cancer: a phase II study Radiother Oncol 2008 89 263 9 10.1016/j.radonc.2008.07.010 18768230

[B15] Seck K Riemer S Kates R Ullrich T Lutz V Harbeck N Schmitt M Kiechle M Diasio R Gross E Analysis of the DPYD gene implicated in 5-fluorouracil catabolism in a cohort of Caucasian individuals Clin Cancer Res 2005 11 5886 92 10.1158/1078-0432.CCR-04-1784 16115930

[B16] Diasio RB Johnson MR Dihydropyrimidine dehydrogenase: its role in 5-fluorouracil clinical toxicity and tumor resistance Clin Cancer Res 1999 5 2672 3 10537327

[B17] Saif MW Diasio R Is capecitabine safe in patients with gastrointestinal cancer and dihydropyrimidine dehydrogenase deficiency Clin Colorectal Cancer 2006 5 359 62 10.3816/CCC.2006.n.007 16512996

[B18] Van Kuilenburg ABP Abreu R Van Gennip A Pharmacogenetic and clinical aspects of dihydropyrimidine dehydrogenase deficiency Ann Clin Biochem 2003 40 41 5 10.1258/000456303321016150 12542909

[B19] Miller AB Hoogstraten B Staquet M Winkler A Reporting results of cancer treatment Cancer 1981 47 207 14 10.1002/1097-0142(19810101)47:1<207::AID-CNCR2820470134>3.0.CO;2-6 7459811

[B20] Mornex F Pavy JJ Denekamp J Bolla M Scoring system of late effects of radiations on normal tissues: the SOMA-LENT scale Cancer Radiother 1997 1 622 68 article in French 9614880 10.1016/s1278-3218(97)82941-1

[B21] Sobin LH Wittekind CH TNM Classification of Malignant Tumours (5th ed) 1997 Wiley-Liss; New York

[B22] Meterissian S Skibber J Rich T Roubein L Ajani J Cleary K Ota DM Patterns of residual disease after pre-operative chemoradiation in ultrasound T3 rectal carcinoma Ann Surg Oncol 1994 1 111 6 10.1007/BF02303553 7834435

[B23] Minsky BD Cohen AM Enker WE Paty P Sphincter preservation with preoperative radiation therapy and coloanal anastomosis Int J Radiat Oncol Biol Phys 1995 31 553 9 10.1016/0360-3016(94)00375-U 7852119

[B24] Morel A Boisdron-Celle M Fey L Soulié P Craipeau MC Traore S Gamelin E Clinical relevance of different dihydropyrimidine dehydrogenase gene single nucleotide polymorphisms (SNP) on 5-fluorouracil tolerance Mol Cancer Ther 2006 5 2895 904 10.1158/1535-7163.MCT-06-0327 17121937

[B25] Remaud G Boisdron-Celle M Hameline C Morel A Gamelin E An accurate dihydrouracil/uracil determination using improved high performance liquid chromatography method for preventing fluoropyrimidines-related toxicity in clinical practice J Chromatogr B Analyt Technol Biomed Life Sci 2005 823 98 107 10.1016/j.jchromb.2005.05.044 16027050

[B26] Boisdron-Celle M Remaud G Traore S Poirier AL Gamelin L Morel A Gamelin E 5-Fluorouacil-related severe toxicity: A comparison of different methods for the pretherapeutic detection of dihydropyrimidine dehydrogenase deficiency Cancer Lett 2007 249 271 82 10.1016/j.canlet.2006.09.006 17064846

[B27] Gamelin E Boisdron-Celle M Guérin-Meyer V Delva R Lortholary A Genevieve F Larra F Ifrah N Robert J Correlation between uracil and dihydrouracil plasma ratio, fluorouracil (5-FU) pharmacokinetic parameters, and tolerance in patients with advanced colorectal cancer: A potential interest for predicting 5-FU toxicity and determining optimal 5-FU dosage J Clin Oncol 1999 17 1105 10 10561167 10.1200/JCO.1999.17.4.1105

[B28] Simon R Optimal two-stage designs for phase II clinical trials Control Clin Trials 1989 10 1 10 10.1016/0197-2456(89)90015-9 2702835

[B29] Kim DY Jung KH Kim TH Kim DW Chang HJ Jeong JY Kim YH Son SH Yun T Hong CW Sohn DK Lim SB Choi HS Jeong SY Park JG Comparison of 5-fluorouracil/leucovorin and capecitabine in preoperative chemoradiotherapy for locally advanced rectal cancer Int J Radiat Oncol Biol Phys 2007 67 378 84 10.1016/j.ijrobp.2006.08.063 17097835

[B30] Dunst J Debus J Rudat V Wulf J Budach W Hoelscher T Reese T Mose S Roedel C Zuehlke H Hinke A Neadjuvant capecitabine combined with standard radiotherapy in patients with locally advanced rectal cancer : mature results of a phase II trial Strahlentther Onkol 2008 184 450 6 10.1007/s00066-008-1751-4 19016023

[B31] Sauer R Becker H Hohenberger W Rödel C Wittekind C Fietkau R Martus P Tschmelitsch J Hager E Hess CF Karstens JH Liersch T Schmidberger H Raab R German Rectal Cancer Study Group Preoperative chemoradiotherapy for rectal cancer N Eng J Med 2004 351 17316 40 10.1056/NEJMoa040694 15496622

[B32] Calvo FA Serrano FJ Diaz-González JA Gomez-Espi M Lozano E Garcia R de la Mata D Arranz JA García-Alfonso P Pérez-Manga G Álvarez E Improved incidence of pT0 downstaged surgical specimens in locally advanced rectal cancer (LARC) treated with induction oxaliplatin plus 5-fluorouracil and preoperative chemoradiation Ann Oncol 2006 17 1103 10 10.1093/annonc/mdl085 16670204

[B33] Ryan DP Niedzwiecki D Hollis D Mediema BE Wadler S Tepper JE Goldberg RM Mayer RJ Phase I/II study of preoperative oxaliplatin, fluorouracil, and external-beam radiation therapy in patients with locally advanced rectal cancer: Cancer and Leukemia Group B 89901 J Clin Oncol 2006 24 2557 62 10.1200/JCO.2006.05.6754 16636336

[B34] Sebag-Montefiore D Glynne-Jones R Falk S Meadows HM Maughan T A phase I/II study of oxaliplatin when added to 5-fluorouracil and leucovorin and pelvic radiation in locally advanced rectal cancer: a Colorectal Clinical Oncology Group (CCOG) study Br J Cancer 2005 93 993 8 10.1038/sj.bjc.6602818 16249791 PMC2361684

[B35] Janjan NA Khoo VS Abbruzzese J Pazdur R Dubrow R Cleary KR Allen PK Lynch PM Glober G Wolff R Rich TA Skibber J Tumor downstaging and sphincter preservation with preoperative chemoradiation in locally advanced rectal cancer: the M. D. Anderson Cancer Center experience Int J Radiat Oncol Biol Phys 1999 44 1027 38 10.1016/S0360-3016(99)00099-1 10421535

[B36] Chan AK Wong A Jenken D Heine J Buie D Johnson D Post-treatment TNM staging is a prognostic indicator of survival and recurrence in tethered or fixed rectal carcinoma after preoperative chemotherapy and radiotherapy Int J Radiat Oncol Biol Phys 2005 61 665 77 10.1016/j.ijrobp.2004.06.206 15708244

[B37] Mawdsley S Glynne-Jones R Grainger J Richman P Makris A Harrison M Ashford R Harrison RA Osborne M Livingstone JI MacDonald P Mitchell IC Meyrick-Thomas J Northover JM Windsor A Novell R Wallace M Can histopathologic assessment of circumferential margin after preoperative pelvic chemoradiotherapy for T3-T4 rectal cancer predict for 3-year disease-free survival? Int J Radiat Oncol Biol Phys 2005 63 745 52 10.1016/j.ijrobp.2005.03.003 16199310

[B38] Muthusamy VR Chang KJ Optimal methods for staging rectal cancer Clin Cancer Res 2007 13 6877 84 10.1158/1078-0432.CCR-07-1137 18006793

[B39] Rasheed S Guenther T Talbot I McDonald P Northover J Stirling J Culver L Glynne-Jones R Padhani AR USPIO -- enhanced rectal cancer specimen MRI: how well does it correlate with node identification at histopathology? Colorectal Dis 2006 8 721 10.1111/j.1463-1318.2006.01102_1.x

[B40] Gérard JP Conroy T Bonnetain F Bouché O Chapet O Closon-Dejardin MT Untereiner M Leduc B Francois E Maurel J Seitz JF Buecher B Mackiewicz R Ducreux M Bedenne L Preoperative radiotherapy with or without concurrent fluorouracil and leucovorin in T3-4 rectal cancers: results of FFCD 9203 J Clin Oncol 2006 24 4620 5 17008704 10.1200/JCO.2006.06.7629

[B41] Bosset JF Collette L Calais G Mineur L Maingon P Radosevic-Jelic L Daban A Bardet E Beny A Ollier JC EORTC Radiotherapy Group Trial 22921 Chemotherapy with preoperative radiotherapy in rectal cancer N Engl J Med 2006 355 1114 23 10.1056/NEJMoa060829 16971718

[B42] Rodríguez-Ramírez SE Uribe A Ruiz-García EB Labastida S Luna-Pérez P Risk factors for anastomotic leakage after preoperative chemoradiation therapy and low RA with total mesorectal excision for locally advanced rectal cancer Rev Invest Clin 2006 58 204 10 16958295

[B43] Urso E Serpentini S Pucciarelli S De Salvo GL Friso ML Fabris G Lonardi S Ferraro B Bruttocao A Aschele C Nitti D Complications, functional outcome and quality of life after intensive preoperative chemoradiotherapy for rectal cancer Eur J Surg Oncol 2006 32 1201 8 10.1016/j.ejso.2006.07.003 16872799

[B44] Chen DJ Nirodi CS The epidermal growth factor receptor: a role in repair of radiation-induced DNA damage Clin Cancer Res 2007 13 6555 60 10.1158/1078-0432.CCR-07-1610 18006754

[B45] Bosset JF Nguyen F Bosset M Servagi-Vernat S Schipman B Recent advances in the treatment of localized rectal cancer Curr Oncol Rep 2008 10 220 4 10.1007/s11912-008-0034-7 18765152

